# Diet quality and depressive symptoms in older adults, assessing the effect modification by genetic predisposition and low-grade inflammation: a target trial emulation

**DOI:** 10.1038/s41380-026-03762-6

**Published:** 2026-07-17

**Authors:** Belayneh Mengist, Najmeh Davoodian, Mojtaba Lotfaliany, Julie A. Pasco, Bruno Agustini, Malcolm Forbes, Wolfgang Marx, Chenglong Yu, Johannes T. Neumann, Paul Lacaze, Alice J. Owen, John J. McNeil, Robyn L. Woods, Michael Berk, Mohammadreza Mohebbi

**Affiliations:** 1 Deakin Institute for Mental and Physical Health and Clinical Translation (Deakin IMPACT), School of Medicine, Deakin University and Barwon Health, Geelong, VIC Australia; 2 College of Medicine and Health Sciences, Debre Markos University, Debre Markos, Ethiopia; 3 School of Public Health and Preventive Medicine, Monash University, Melbourne, VIC Australia; 4 Department of Cardiology, University Heart & Vascular Center, Hamburg, University Medical Center Hamburg-Eppendorf, Hamburg, Germany; 5 German Center for Cardiovascular Research (DZHK), Partner Site, Hamburg/Kiel/Lübeck, Hamburg, Germany; 6 Biostatistics Unit, Faculty of Health, Deakin University, Geelong, VIC Australia

**Keywords:** Depression, Genetics

## Abstract

Longitudinal studies have shown an association between diet quality and depression. However, reverse causality, unmeasured confounding, and selection bias remained important limitations. We aim to examine the relationship between diet quality and depression in older adults while addressing these issues and explore modification role of genetic predisposition to depression and low-grade inflammation. We emulated a target trial of dietary interventions using data from the ASPREE cohort. An ultra-processed food (UPF) index and an anti-inflammatory diet measure were extracted from a food frequency questionnaire to quantify diet quality. Depressive symptoms were assessed annually with a Center for Epidemiologic Studies-Depression 10-item score of ≥8. A polygenic score was derived using the latest Psychiatric Genomics Consortium data for major depression. Systemic inflammation was assessed using circulating high-sensitivity C-reactive protein. Inverse probability treatment weighting was applied to balance measured confounders. The effects of diet quality on depressive symptoms were estimated using generalised estimating equations. A total of 7220 participants (52.7% female), aged 70+ years, were followed for a median of 5.7 years. High UPF consumption was associated with a higher risk of depressive symptoms (RR: 1.12, 95% CI: 1.03–1.21), while an anti-inflammatory diet was associated with lower depressive symptoms (RR: 0.93, 95% CI: 0.86–1.00). Genetic predisposition or low-grade inflammation did not modify the observed associations. Higher diet quality is associated with a lower risk of depressive symptoms, independent of genetic predisposition or low-grade inflammation, which may support dietary interventions as a modifiable lifestyle strategy for mental health promotion and prevention in older adults.

## Introduction

Late-life depression is a common and debilitating mental health condition that affects many older adults [1]. A recent meta-analysis reported that approximately 19% of older adults worldwide experience depressive symptoms [2]. Late-life depression is associated with higher rates of suicidality, medical and psychiatric comorbidities, poorer quality of life, and greater burden on health systems and caregivers [3, 4]. It is the second leading cause of disability [4].

Depression arises from complex interactions of genetic, psychosocial, lifestyle, and environmental factors [5]. Evidence shows that genetic predisposition plays a significant role in the development of various mental disorders, including depression [6]. Genome-wide association studies (GWAS) indicate that genetic liability increases the risk of depression [7]. Although individuals may have a greater genetic susceptibility to depression, their overall risk is also modulated by environmental exposures and lifestyle factors [5].

Low-grade systemic inflammation significantly contributes to the development and progression of depression [8]. Elevated inflammatory markers, particularly C-reactive protein (CRP), are associated with increased risk and severity of depressive symptoms, with high-sensitivity CRP serving as a predictor of depression [9, 10]. Studies also show that people with depression exhibit low-grade inflammation, reflected by elevated CRP levels [11].

Lifestyle factors, including diet quality, physical activity, smoking, and social support, are established determinants of depressive symptoms [12, 13]. Among these factors, diet quality is a particularly important and modifiable target, with effects that may extend beyond general physical health to biological pathways implicated in depression [14]. This evidence has contributed to the emergence of nutritional psychiatry, a field that examines how diet and nutrients influence mental and brain health through mechanisms such as neuroinflammation, oxidative stress, gut-brain interactions, and neurotransmitter metabolism, with the broader aim of integrating nutrition into psychiatric prevention and care [15]. A healthy dietary pattern, such as the Mediterranean diet, an anti-inflammatory diet, or adherence to a higher healthy eating index, is associated with reduced depression risk [16]. In contrast, a low-quality diet, such as ultra-processed food and a pro-inflammatory diet, is associated with an increased risk of depression [16–18].

Most published studies assessing the relationship between diet and depression failed to account for genetic susceptibility or systemic inflammation and are prone to reverse causality bias [19]. It is also established that genetic factors influence food intake and dietary pattern selection [20, 21] and that systemic inflammation may mediate the relationship between diet and depression [22]. Integrating genetic information with observational data improves the performance and reliability of trial emulations and provides potential insights into residual confounding [23]. In addition, whether genetic susceptibility or low-grade systemic inflammation moderates the association between diet and depression is poorly understood. A cohort study using data from the UK Biobank examined the interaction between genetic risk for depression and a composite lifestyle score of five lifestyle factors, including diet; however, diet quality was not assessed independently [24].

Conducting a randomised controlled trial to investigate the effect of a diet intervention on mental health outcomes in older adults is challenging due to limitations related to ethics, adherence to an interventional diet over a long period, blinding of the intervention, the risk of consequence-expectation bias, cost, and feasibility [25]. Thus, we applied a target trial emulation to investigate the effect of diet quality using two complementary measures, ultra-processed food (UPF) consumption and the dietary inflammation score on depressive symptoms, adjusting for a wide range of confounders, including sociodemographic characteristics, morbidities, medication use, lifestyle factors, genetic susceptibility, and inflammatory status, using a large cohort of older adults in Australia. Moreover, we investigated the potential modifying effect of genetic susceptibility and inflammatory status on the association between diet quality and depressive symptoms. In addition, we aimed to explore the role of genetic susceptibility in sex-specific associations between diet and depression. Unlike genes, diet is a modifiable risk factor for depression. Thus, this study can identify subgroups that may benefit most from targeted dietary interventions and provide evidence supporting precision medicine.

## Methods

### Study design and population

We designed a target trial emulation using longitudinal data from the ASPREE-eXTension (ASPREE-XT) cohort study [26]. ASPREE-XT cohort is a prospective observational follow-up (2018–2024) of over 15,000 older adults from the original ASPirin in Reducing Events in the Elderly (ASPREE) study. ASPREE was a double-blind, randomised, placebo-controlled trial aimed to investigate the effect of daily 100 mg enteric-coated aspirin on disability-free survival in 19,114 individuals aged 70+ years from Australia and 65+ years from the US. Participants were free from overt cardiovascular disease, dementia, major physical disability, and a 5-year life-limiting disease at enrolment and were recruited from 2010 to 2014. The trial was completed in June 2017. Details of the sample, study design, recruitment, eligibility criteria, and baseline participant characteristics are provided elsewhere [26, 27].

Annual depressive symptoms, as measured by the 10-item Center for Epidemiologic Studies Depression Scale (CES-D), were collected in the Aspirin for the Prevention of Depression in the Elderly (ASPREE-D) study, a sub-study of ASPREE that investigated the effect of low-dose aspirin on depression [28]. ASPREE-D showed that low-dose aspirin did not prevent depression in otherwise healthy older adults and did not improve the long-term course of depressive symptoms among those with pre-existing depression [29, 30]. Data for diet quality were taken from the ASPREE Longitudinal Study of Older Persons (ALSOP), a sub-study of ASPREE among Australian participants, commenced in early 2012 [31]. Genetic and biomarker (hs-CRP) data were drawn from the ASPREE Biobank. This sub-study included Australian participants who provided written consent for their samples to be used for biomarker and/or genetic analysis. Participants provided samples within 12 months of randomisation. The methodology and participant characteristics of the ASPREE Healthy Ageing Biobank have been reported previously [32].

**Fig. 1:**
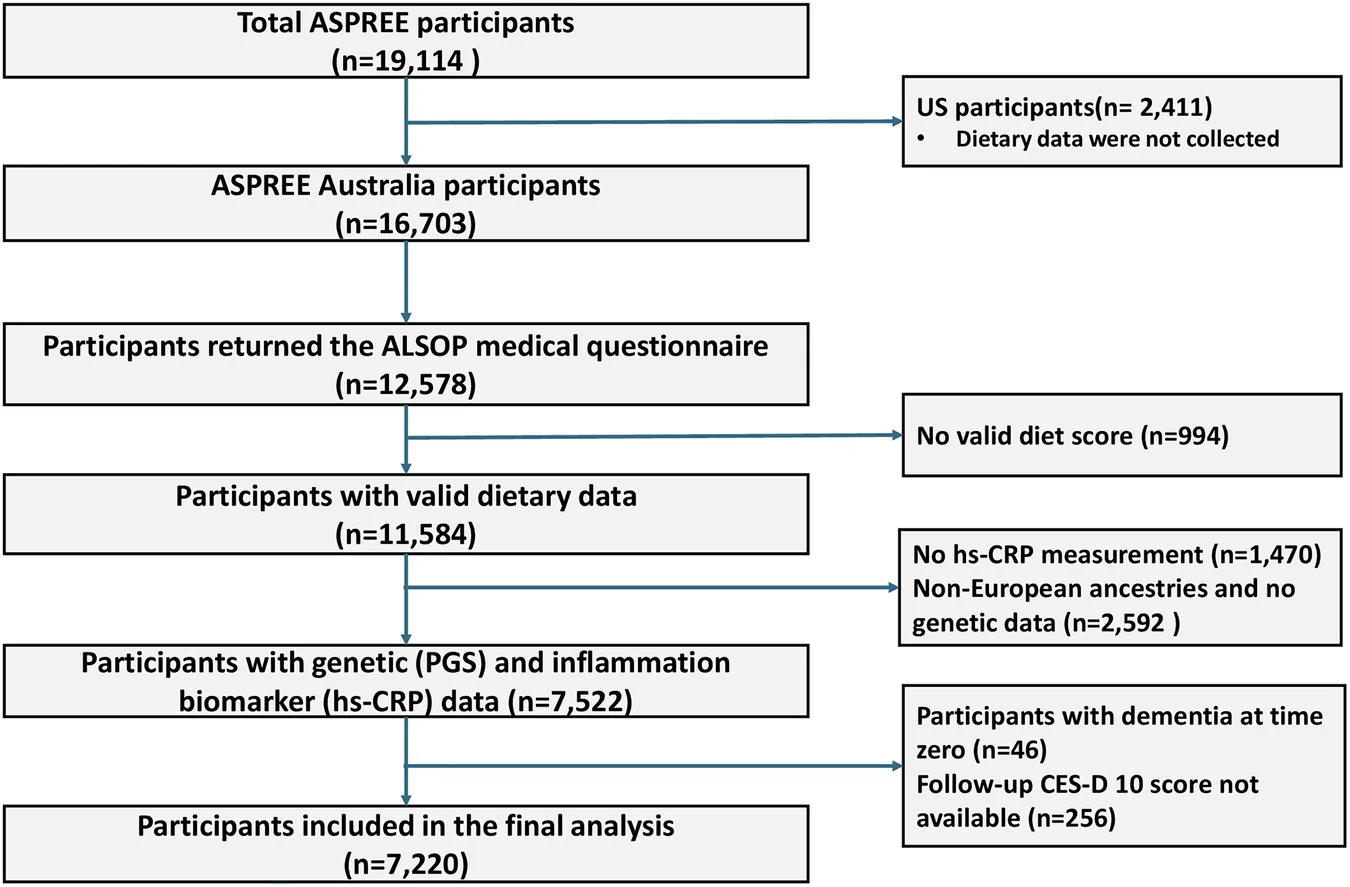
Study flowchart showing participants included in the study.

In this study, we included participants with valid ALSOP dietary data, genotyped participants of European descent, and who had hs-CRP biomarker data and at least one CES-D-10 measurement during follow-ups through 2022. We considered the ASPREE year 3 data collection visit, which approximately corresponds to the timing of the ALSOP dietary questionnaire, as time zero. The study flow chart is presented in Fig. 1.

### Target trial specification and emulation

We applied the design principles of the target trial methodology to estimate the effect of hypothetical pragmatic dietary interventions on depressive symptoms. Using the target trial emulation framework, we developed a hypothetical pragmatic target trial protocol that included eligibility criteria, intervention strategy, intervention assignment, outcomes, follow-up, causal contrast and statistical analysis. The protocol was then emulated using the ASPREE extended follow-up longitudinal observational data, following the Transparent Reporting of Observational Studies Emulating a Target Trial (TARGET) guideline. The protocol details are provided in Table 1.

**Table 1:** Design and emulation of a target trial of diet interventions on depressive symptoms using ASPREE longitudinal data.

Protocol component	Target trial specification	Target trial emulation
Eligibility criteria	Community-dwelling older adults 70 years and older in Australia. **Exclusion criteria:** • Dementia at time zero • Non-European descent	Same as the target trial. **In sensitivity analyses:** • Participants with clinically relevant depressive symptoms (CES-D 10 ≥ 8) at time zero were excluded • Participants taking antidepressants (ATC code N06A) at time zero were excluded
Diet strategy	**Strategy one** • Random allocation to high ultra-processed food (UPF) consumption • Control: low UPF consumption **Strategy two** • Random allocation to an anti-inflammatory diet • Control: pro-inflammatory diet	• UPF consumption was determined based on the NOVA classification. • High UPF consumption was defined as ≥4 servings/day, and low UPF consumption (<4 servings/day). • Inflammation potential of diet was defined based on the dietary inflammation score (DIS); an anti-inflammatory diet <0 and a pro-inflammatory diet >0 of the score.
Intervention assignment	Participants were randomly assigned to diet strategies at time zero and were aware of the dietary strategy they received (randomisation, but no blinding).	Randomisation was mimicked by applying inverse probability treatment weighting (IPTW) to balance measured confounders, including sociodemographic, morbidities, medication use, biomarkers, genetic susceptibility and inflammatory status.
Outcomes	Presence of depressive symptoms	Same as the target trial. We defined depressive symptoms as the presence of clinically relevant depressive symptoms measured by a total score of ≥8 on the CES-D 10 scale at the follow-ups. Outcome was considered one year after time zero to allow a grace period between intervention and outcome, while balancing the groups on the time zero CES-D 10 score.
Follow-up	Participants’ annual follow-up after randomisation (intervention assignment) until lost follow-up, death, or end of the study (2022).	Same as the target trial. Time zero was defined as year 3 of the ASPREE data collection visit, when the diet questionnaire was administered, and continued through the last available follow-up of ASPREE-XT04 (January 2022).
Causal contrast	Intention-to-treat effect of the allocated intervention strategies (high vs low UPF consumption and anti-inflammatory vs pro-inflammatory diet)	Intention-to-treat effect (effects of interventions as specified in the protocol) and counterfactual contrasts (potential outcomes) were estimated. Causal contrasts for interventions were quantified using risk ratios.
Statistical analysis	Intent-to-treat analysis using generalised estimating equation models to incorporate CES-D 10 repeated measures at annual follow-ups. The effect modifications by genetic susceptibility for depression and inflammatory status were investigated. Prespecified subgroup and sensitivity analyses were conducted, stratified by sex (male/female), the CES-D 10 cutoff (≥10), and polygenic score categories (low, medium, and high).	Intent-to-treat analysis using weighted generalised estimating equation models. A marginal structural model was used, with IPTW calculated from propensity scores. Missing covariate data were handled using predictive mean matching. Effect modification by genetic susceptibility for depression and inflammatory status was investigated. Same as the target trial

### Outcome

Depressive symptoms were assessed using the CES-D 10 scale. The CES-D 10 is a validated instrument for assessing clinically relevant depressive symptoms. It consists of a 10-item self-administered questionnaire, with total scores ranging from 0 to 30, where higher scores indicate greater severity of depressive symptoms [33, 34]. Clinically relevant depressive symptoms were defined as a CES-D 10 score of ≥8, which was labelled as depressive symptoms in this study [35]. We also used a CES-D 10 cutoff score of ≥10 to represent a higher threshold for clinically significant depressive symptoms. CES-D 10 scores after year 3 ASPREE data collection visit through ASPREE-XT04 (year 4 to year 11) were included as a repeatedly measured outcome, while the CES-D 10 score at time zero (year 3) was balanced between the intervention groups (S. Fig. 1).

### Polygenic score for depression

We utilised the previously generated polygenic score (PGS) for major depression in the ASPREE cohort. DNA samples from the ASPREE Biobank were genotyped with the Axiom 2.0 Precision Medicine Diversity Research Array and imputed using the TOPMed Imputation Server (TOPMed-r2 panel). PGS were derived from ~7 million single-nucleotide polymorphisms (SNPs) using weights estimated by the SBayesRC method [36] applied to the latest Psychiatric Genomics Consortium (PGC) GWAS summary statistics [37]. The details of the genotyping, quality control, imputation, and PGS derivation procedures are described elsewhere [38]. PGS for major depression was categorised into low (quintile one, ≤20%), medium (quintiles 2 to 4, 21–80%), and high (quintile 5, >80%) utilising similar approaches as described previously [38].

### Inflammatory biomarkers

High-sensitivity CRP was measured in non-fasting blood samples collected at baseline, during the ASPREE clinical trial recruitment period, and approximately at year 3 of the trial, as part of the ASPREE Biobank sub-study. All plasma samples were stored at −80 °C and analysed in Hamburg, Germany. Hs-CRP concentrations (mg/L) were quantified using the ARCHITECT i2000SR immunoassay system with average coefficients of variation (CV%) of 1.0% at 3.4 mg/L, 1.5% at 9.8 mg/L, and 2.8% at 28.6 mg/L [39]. The details of sample collection, processing, storage and curation can be found elsewhere [32]. Low-grade inflammation was defined as hs-CRP > 3 mg/L [40]. Both the categorical and log-transformed hs-CRP were used in the analysis. In this analysis, we used the year 3 hs-CRP measurement.

### Diet quality

Two indicators of diet quality were derived from the diet screener questionnaire: ultra-processed food (UPF) consumption and the dietary inflammation score (DIS).

### Dietary assessment

Dietary information was assessed using a self-administered 54-item food frequency questionnaire embedded within the ALSOP sub-study medical questionnaire, collected approximately at the year-3 ASPREE data collection visit [31]. The questionnaire measured participants’ dietary intake, including food and drink items categorised into food groups such as meat, fish and eggs; snack and convenience foods; dairy (including milk and milk alternatives); bread, grains and cereals; fruit and vegetables; drinks (soft drinks, cordial, supplement drinks, etc.); and other nutrients (salt, fats and oils, water, and discretionary foods). Consumption frequency was self-reported for the last 12 months of the diet, measured on a scale ranging from “never” to “every day/several times a day” (S. Table 1).

Data from the diet questionnaire were converted to a daily equivalent frequency for each food, and serving frequency per day was computed for each participant by summing the daily equivalent score of each food item, as described in detail previously [18].

Data on micronutrient supplements, including vitamins B, C, D, and E, multivitamins, calcium, and zinc, were collected through self-reported questions and recorded as none, occasionally, or daily.

### Ultra-processed food (UPF) consumption

The degree of food processing was classified based on the NOVA food classification system [41]. Foods and drinks on the diet screen questionnaire were classified into four groups (unprocessed/minimally processed, culinary ingredients, processed foods, and ultra-processed foods). Twenty-one food and drink items were classified as NOVA group 4 (UPF). Daily UPF consumption was calculated as servings per day by converting UPF frequency from the diet screener. UPF consumption was a-priori categorised as high (≥4 servings/day) and low (<4 servings/day) [42]. The details of the UPF consumption score have been described previously [18].

### Dietary Inflammation Score (DIS)

The DIS was calculated according to the methods described by Byrd et al. [43]. Food groups and supplements were categorised into 18 components (17 food groups and one supplement group) as described previously [44]. The food groups include leafy greens and cruciferous vegetables, deep yellow or orange vegetables and fruit, apples and berries, other fruits and real fruit juices, fish, legumes, poultry, red and organ meats, processed meats, refined grains and starchy vegetables, high-fat dairy, low-fat dairy, coffee and tea, other fats, added sugars and nuts. Tomatoes were collected with fruits/vegetables from the food screening questionnaire and were not recorded separately. Supplement (minerals and vitamins) intake was assessed based on utilisation frequency: 0 = none, 1 = occasional use, and 2 = everyday use, and a total supplement score was derived by summing each micronutrient value. The study sample was standardised for each component (17 food groups based on servings and one supplement score) by sex, to a mean of 0 and unit standard deviation. The DIS was calculated by multiplying the food group and supplement by their respective weights of inflammatory score, as outlined by Byrd et al., and the weighted values were summed to form the DIS [43]. The DIS was developed by assessing the strengths of the multivariable-adjusted associations of each food group with measured circulating biomarkers of inflammation [43]. Finally, DIS was categorised into anti-inflammatory (DIS < 0) and pro-inflammatory (DIS > 0) for the analysis (no one with DIS = 0). The positive score indicates pro-inflammatory potential and the negative score indicates anti-inflammatory potential of the diet; details of the scoring methods are found elsewhere [43]. The DIS developed by Byrd et al. was validated in three different populations and replicated by several studies [45–47].

Additionally, sex-specific quartiles of both UPF and DIS were used for analysis.

### Confounders and covariates

Covariates include sociodemographic characteristics (age, sex, years of education and income), lifestyle factors (smoking status, alcohol use, intensity of physical activity, and social support), living situation (living alone or with family/in a residential home), body mass index (BMI) in kg/m^2^, CES-D 10 score at time zero, cognitive function (Modified Mini-Mental State examination (3MS)), multimorbidity (coexistence of two or more chronic health conditions), metabolic syndrome (based on the Adult Treatment Panel III (ATP III) diagnostic criteria), anti-inflammatory medication use and polypharmacy (use of five or more prescription medications).

**Fig. 2:**
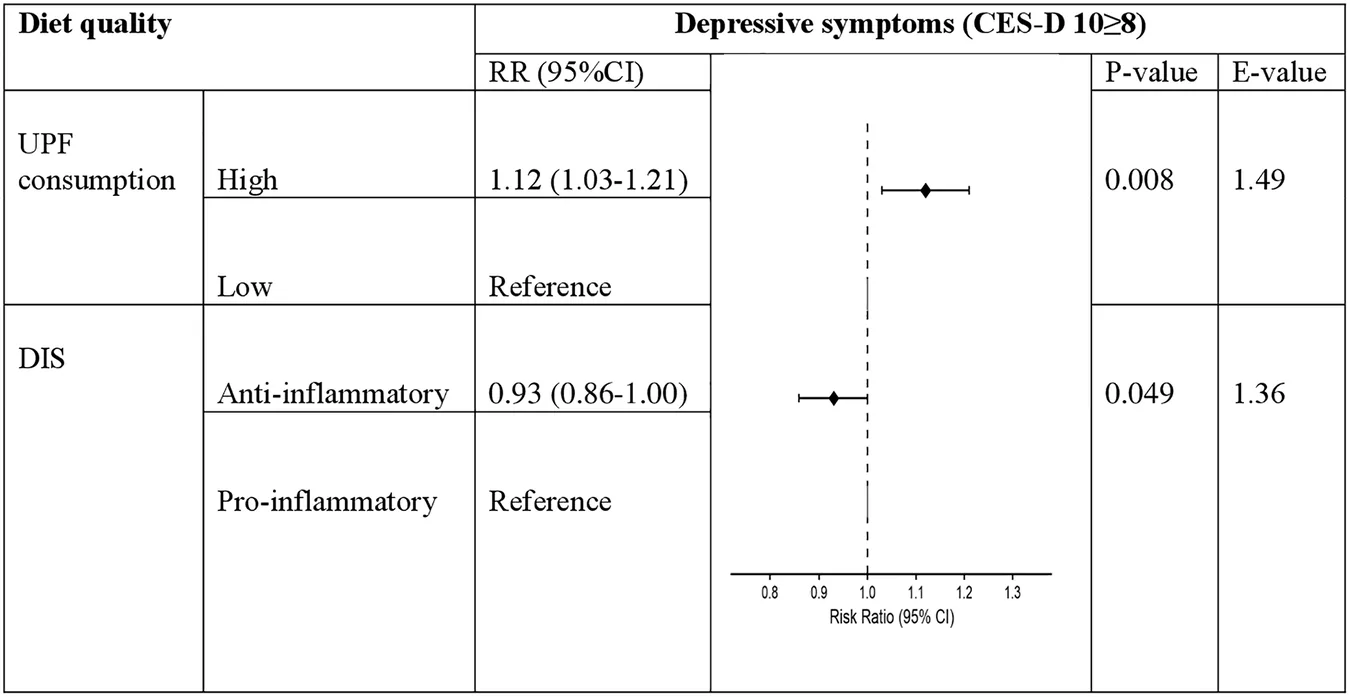
Association between ultra-processed food and diet inflammation score with the risk of depressive symptoms. Covariates included in IPTW: age, sex, education status, alcohol use, smoking, income, social support, living status, physical activity, BMI, metabolic syndrome, cognition status, multimorbidity, polypharmacy, hs-CRP, PGS and CES-D 10 at time zero. Anti-inflammatory medications were included in IPTW for DIS models. E-value: the minimum strength of association on the risk ratio scale that an unmeasured confounder would need to have to explain away the observed effect. A large E-value indicates that a substantial unmeasured confounding effect is needed to nullify the observed effects. In this study, E-values were 1.49 and 1.36 for the UPF and DIS diets, respectively, indicating that the findings were robust to residual confounding bias.

The PGS for depression and the hs-CRP were included as covariates in the primary analysis to assess the association between diet quality and depression and were considered effect modifiers in additional models, as outlined in the statistical analysis section. All covariates are at time zero. We used causal directed acyclic graphs (DAGs) to identify potential confounders (S. Fig. 2).

### Statistical analysis

**Fig. 3:**
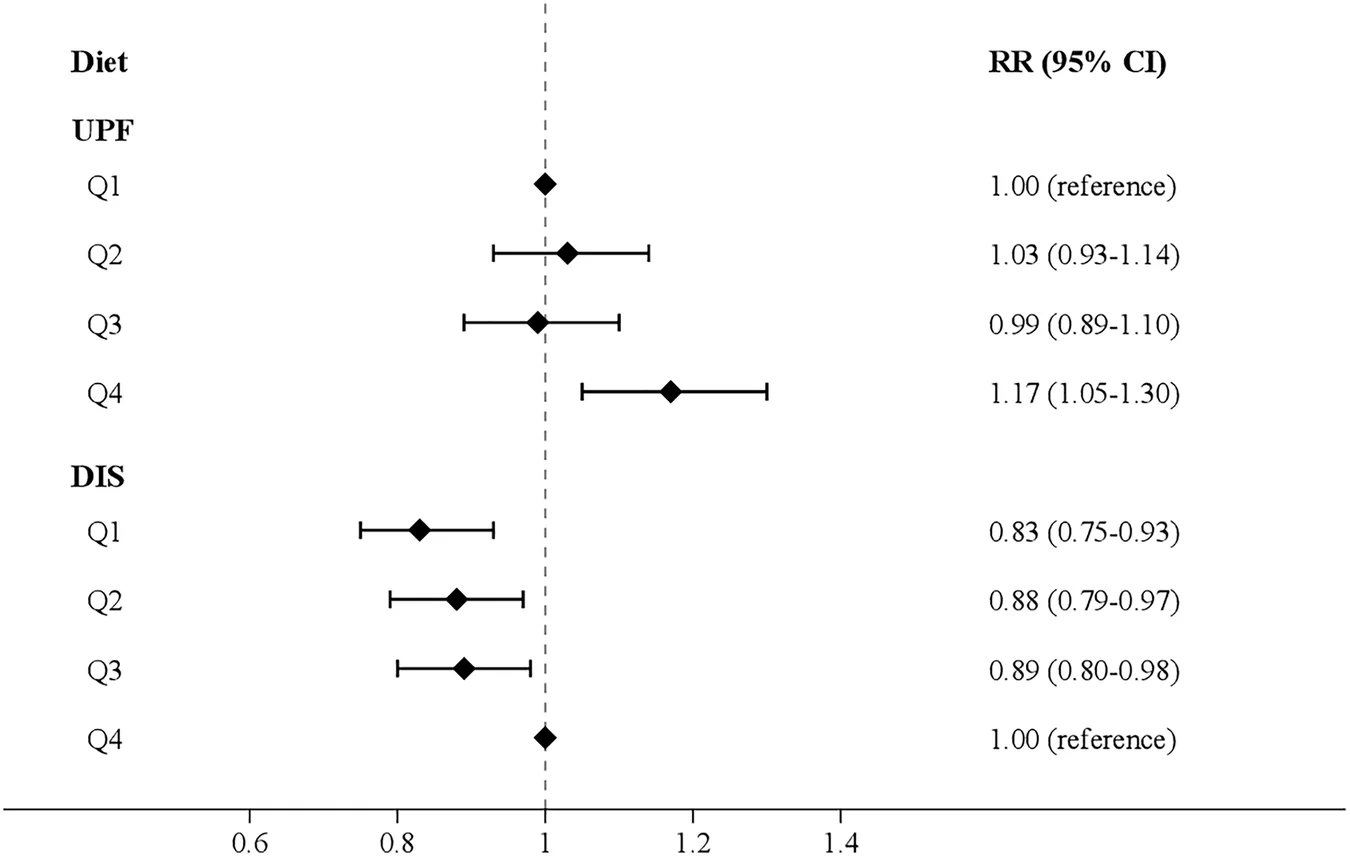
Association between diet quality and the risk of depressive symptoms using quartiles of the diet scores. UPF: Ultra-processed food, Q4 is the highest UPF consumption, DIS: Dietary inflammation score, Q1 is the most anti-inflammatory diet, and Q4 is the most pro-inflammatory diet. Quartiles are sex specific. All covariates accounted for in the main model were used in IPTW.

Participant characteristics were summarised using frequencies and percentages for categorical variables and means with standard deviations for continuous variables, as appropriate. A propensity score was estimated using logistic regression, with diet quality indicators (UPF or DIS) as the outcome, conditional on the prespecified covariates. We applied inverse probability treatment weighting (IPTW) to balance potential confounders and mimic a random assignment of the intervention [48]. Covariate balance in the exposures was assessed before and after IPTW using standardised mean difference (SMD), where a value < 0.1 indicates good balance [49] (S. Figs. 3 and 4). We applied weight truncation at the 2nd and 98th percentiles to avoid bias from the extreme weights [50]. Generalised estimating equations (GEE) models were fitted to the balanced exposure groups (i.e., after employing the IPTW) using a log link function and a first-order autoregressive correlation structure. Separate models were run for each diet exposure (UPF and DIS). The risk of depressive symptoms was compared across the diet exposure groups. E-values were calculated to assess the robustness of observed associations to potential unmeasured confounding. Additionally, a propensity score was estimated using ordinal logistic regression for diet quartiles conditional on the covariates used in the main model, and IPTWs were calculated.

The roles of genetic susceptibility and low-grade systemic inflammation (i.e., hs-CRP) as effect modifiers in the association between dietary quality and depressive symptoms were investigated by estimating two-way interactions between standardised PGS and log-hs-CRP with diet scores (UPF consumption: high vs low; DIS: anti-inflammatory vs pro-inflammatory diet). For the interaction models, new IPTWs were calculated by excluding PGS and hs-CRP. Stratified analyses by PGS categories (low, medium, and high) and by hs-CRP (high and low) were conducted using approaches similar to those used in the main analysis. New IPTWs were calculated for subgroup analyses.

Moreover, a sex-disaggregated analysis was conducted using methods similar to those of the main analyses. Subgroup and interaction analyses were conducted by aspirin (treatment vs placebo) for the ASPREE trial period. Sensitivity analyses were conducted, excluding participants with depressive symptoms (CES-D 10 ≥ 8) and receiving antidepressants at time zero and using the CES-D 10 cut-off ≥10. Risk ratios (RRs) along with their corresponding 95% confidence intervals (CIs) were reported. All tests were two-sided, and statistical significance was determined by a p-value < 0.05. All analyses were performed using R software (*ipw* and *geepack* packages), version 4.3.3 (R Foundation for Statistical Computing).

## Results

### Study population characteristics

A total of 7220 older adults were included in the analysis. The mean (sd) age of participants was 77.6 ± 3.9, and more than half (52.7%) were female. At time zero, 75.8% of participants were current alcohol users, and 63.5% engaged in moderate to vigorous physical activity. Most participants had adequate income (92.6%) and good social support (90.9%). Overall, 81.3% of participants had multimorbidity, and 41.0% used polypharmacy. Of the participants included in the analysis, 30.2 and 44.9% consumed high-UPF and pro-inflammatory diets, respectively (Table 2). The median (IQR) follow-up was 5.7 (2.4) years, and the average number of CES-D 10 repeated measurements over the follow-up period was 5. Based on hs-CRP levels, 28.6% of participants had low-grade inflammation.

**Table 2:** Participant characteristics by diet quality.

Covariates	Diet quality							
	UPF consumption (n = 7220)				Dietary inflammation score (n = 7103)			
	Low (n = 5037)	High (n = 2183)	SMD		Anti-inflammatory (n = 3912)	Pro-inflammatory (n = 3191)	SMD	
			Unweighted	Weighted			Unweighted	Weighted
Age (years): mean (sd)	77.4(3.8)	78.1(4.2)	0.168	0.005	77.5(3.9)	77.8(4.0)	0.092	<0.001
Sex: female	2914(57.9)	891(40.8)	0.346	0.003	2051(52.4)	1673(52.4)	<0.001	0.002
Education: >12 years	2127(42.2)	926(42.4)	0.004	0.005	1755(44.9)	1270(39.8)	0.103	<0.001
Smoking status: yes	124(2.5)	24(1.1)	0.103	0.002	78(2.0)	71(2.2)	0.016	<0.001
Alcohol use: yes	3878(77.0)	1596(73.1)	0.090	0.004	3012(77.0)	2393(75.0)	0.047	0.001
BMI (kg/ m^2^): mean (sd)	27.7(4.5)	27.3(4.3)	0.100	0.008	27.5(4.4)	27.6(4.5)	0.034	0.001
Living status: living at home with family or friends	3363(66.8)	1585(72.6)	0.127	0.011	2607(66.6)	2289(71.7)	0.110	0.001
Physical activity: moderate	3151(62.6)	1390(63.7)	0.023	0.004	2555(65.3)	1942(60.9)	0.092	0.001
Income: adequate	4638(92.1)	2024(92.7)	0.024	0.004	3636(92.9)	2937(92.0)	0.034	0.001
Social Support: good	4570(90.7)	1973(90.4)	0.012	0.003	3566(91.2)	2870(89.9)	0.042	0.001
CES-D 10 score at time zero: mean (sd)	4.0(3.8)	4.2(3.9)	0.057	<0.001	3.8(3.8)	4.2(3.9)	0.111	0.001
Cognitive function (3MS): mean(sd)	94.9(4.2)	94.4(4.4)	0.110	0.002	94.9(4.2)	94.5(4.6)	0.102	<0.001
Polygenic score: mean (sd)	−1.27e-09(2.9e-08)	−2.38e-09(2.9e-08)	0.023	0.010	−1.19^e-09^(2.9^e-08)^	−2.10^e-09^(2.9^e-08)^	0.016	0.001
Log hs-CRP: mean (sd)	0.58(1.03)	0.58(1.05)	0.003	0.008	0.54(1.03)	0.63(1.04)	0.092	0.001
Multimorbidity: yes	4055(80.5)	1789(82.0)	0.037	0.009	3112(79.6)	2640(82.7)	0.081	0.001
Metabolic Syndrome: yes	2783(55.3)	1115(51.1)	0.084	0.004	2078(53.1)	1773(55.6)	0.049	<0.001
Anti-inflammatory medication use: yes	879(17.5)	396(18.1)	0.018	*	699 (17.9)	577(18.1)	0.006	0.001
Polypharmacy: yes	2093(41.6)	865(39.6)	0.039	0.003	1540(39.6)	1359(42.6)	0.066	<0.001

*ATC* The WHO Anatomical Therapeutic Chemical Classification System, *SMD* standardised mean difference, * not included in the UPF model.

• Education: years of education (≤12 years and >12 years), smoking: current smoking status (yes vs no), alcohol: current alcohol use (yes vs no), Income: self-reported (adequate vs low or inadequate).

• Physical activity: usual intensity of physical activity in a typical week; low (never, rare or light) and moderate (moderate to vigorous).

• Social support: defined based on the Lubben Social Network Scale (LSNS-6), and a value of ≥12 out of 30 points was considered good social support.

• CES-D 10: Center for Epidemiologic Studies - Depression 10 scale at time zero (year 3 ASPREE data collection visit).

• Metabolic syndrome: based on the Adult Treatment Panel III (ATP III) diagnostic criteria as a composite measure of 5 variables (waist circumference, blood pressure, triglyceride, high-density lipoprotein cholesterol level and blood glucose).

• Multimorbidity: the coexistence of two or more chronic conditions, including hypertension, diabetes mellitus, dyslipidemia, chronic kidney disease, gastro-oesophageal reflux disease, pulmonary disease, gout, cancer, cardiovascular diseases and Parkinson’s disease.

• Anti-inflammatory medications (ATC code M01A) reported at time zero. ATC code M01A designates non-steroidal anti-inflammatory and antirheumatic products (NSAIDs) for systemic use.

• Polypharmacy: the use of five or more prescription medications.

### Association between diet quality and risk of depressive symptoms

After employing IPTW to balance all measured confounders, high UPF consumption (≥4 servings/day) was associated with a 12% higher rate of depressive symptoms than low UPF consumption (RR: 1.12, 95% CI: 1.03–1.21). The estimated number needed to treat for low UPF compared with high UPF over the study period was 48 (95% CI: 27–167), suggesting that for every 48 older adults who adhere to low UPF, there is an associated reduction of one depressive symptom incidence over the study period. Conversely, an anti-inflammatory diet was associated with a 7% lower risk of depressive symptoms compared to a pro-inflammatory diet (RR: 0.93, 95% CI: 0.86–1.00) (Fig. 2).

In quartile-based analysis, quartile 4 (the highest UPF consumption) was associated with a 17% higher rate of depressive symptoms than quartile 1 (the lowest UPF consumption) (RR: 1.17, 95% CI: 1.05–1.30), p-for trend = 0.012. Conversely, for DIS, quartile 1 (the most anti-inflammatory diet) was associated with a 17% lower risk of depressive symptoms compared with quartile 4 (the most pro-inflammatory diet) (RR: 0.83, 95% CI: 0.75–0.93), p-for trend = 0.001. In addition, the association between DIS and depressive symptoms reveals a dose-response pattern (Fig. 3). The estimated number needed to treat for an anti-inflammatory diet compared with a pro-inflammatory diet (Q1 vs. Q4) over the study period was 31 (95% CI: 18–74), suggesting that for every 31 older adults who adhere to an anti-inflammatory diet, there is an associated reduction of one depressive symptom incidence over the study period.

### Effect modification assessment of PGS and hs-CRP

Two-way interaction between polygenic score for major depression and diet quality was not significant, indicating no evidence of effect modification by genetic susceptibility for depression in the association between diet quality and depressive symptoms. Similarly, we found no significant interaction between hs-CRP and diet quality, suggesting that low-grade systemic inflammation did not modify the relationship between diet quality and depressive symptoms (S. Table 2 and S. Fig. 5). Interaction effects for the categorical PGS or hs-CRP were explored and remained non-significant (S. Table 3).

In exploratory stratified analyses by PGS category, the association between UPF consumption and depressive symptoms was significant in the low PGS category. Among participants in the low PGS category, consumption of high UPF was associated with a 25% increased risk of depressive symptoms compared to low UPF consumption (RR:1.25, 95% CI: 1.03–1.52). On the other hand, among participants in the medium PGS category, an anti-inflammatory diet was associated with an 11% lower risk of depressive symptoms than a pro-inflammatory diet (RR: 0.89, 95% CI: 0.81–0.98) (S. Fig. 6). When we combined genetic risk and diet quality scores, strong associations were observed in groups with high genetic risk and high UPF intake, as well as in those with low genetic risk and an anti-inflammatory diet (S. Fig. 7).

### Exploratory analyses

The interaction between sex and diet quality was not significant for either diet score (p for interaction: UPF = 0.32 and DIS = 0.38). In exploratory sex-disaggregated analyses, the association between diet quality and depressive symptoms was significant among female participants, whereas no association was observed among male participants (S. Table 4). Additionally, we conducted further exploratory effect modification analyses of genetic predisposition and low-grade inflammation by sex groups. The interaction between diet quality and PGS for depression was significant only in males for both diet scores (S. Table 5 and S. Fig. 8).

There was no significant interaction between allocation to aspirin or placebo and either diet quality score (p for interaction: UPF = 0.51; DIS = 0.23). In exploratory stratified analyses by aspirin treatment, we found a significant association with UPF consumption only in the aspirin group (S. Table 6).

### Sensitivity analysis

We conducted sensitivity analyses excluding participants with depressive symptoms (n = 1139) and participants who were on antidepressants/antipsychotics (n = 1043) at time zero, with findings consistent with the main analysis (S. Table 7).

A sensitivity analysis using the CES-D 10 cut-off ≥10 indicated that high UPF consumption was associated with a 11% higher risk of depressive symptoms than low UPF consumption (RR: 1.11, 95% CI: 1.00–1.24). While a similar trend was observed between an anti-inflammatory diet and depressive symptoms (RR: 0.93, 95% CI: 0.84–1.02), the association was not significant (S. Table 8). No significant effect modification was observed in interaction analyses.

## Discussion

This longitudinal study of more than 7200 older adults aged 70 or older found that diet quality was associated with depressive symptoms after controlling for a range of pre-specified confounders. High UPF consumption was associated with a higher risk of depressive symptoms, while an anti-inflammatory diet was associated with a lower risk of depressive symptoms. High UPF consumption was associated with a 12% higher rate of depressive symptoms, whereas adherence to a low-UPF consumption (<4 servings/day) was associated with a reduction of approximately one depressive symptom for every 48 older adults over the study period. The associated protective effect of better diet quality against depression remained consistent irrespective of genetic susceptibility for depression or systemic inflammation status, suggesting that dietary modifications may aid in the prevention of depressive symptoms in older populations, regardless of an individual’s genetic risk profile and inflammatory status. By employing a target trial emulation framework with comprehensive confounding adjustment, including demographic, lifestyle, clinical factors, genetic predisposition and inflammatory status, along with an extended follow-up period, this study addresses a critical methodological gap in the existing literature, including the issue of reverse causality.

Our findings align with a substantial body of evidence indicating that adherence to a high-quality diet reduces the risk of depression. This association has been documented in previous studies [16, 19, 51, 52], suggesting that dietary improvement may serve as a modifiable preventive strategy for depressive symptoms. A high-quality diet characterised by greater intake of fruits, vegetables, whole grains, and dietary fibre plays a critical role in reducing systemic inflammation and oxidative stress and supports the gut microbiome balance [22, 53]. These factors are increasingly recognised as potential mechanistic pathways in the development and progression of depressive symptoms. In contrast, unhealthy dietary patterns such as consumption of UPF and a pro-inflammatory diet are associated with increased inflammatory responses, oxidative stress, gut microbiota dysregulation, and mitochondrial dysfunction [22, 54]. Such biological disturbances can adversely affect neuroimmune and neuroendocrine systems, eventually increasing the risk of depression [5, 22]. However, in our study, the attenuation of observed associations at the higher CES-D 10 threshold likely reflects greater clinical heterogeneity among individuals with more severe symptoms, as well as a diminished relative contribution of diet within more complex depressive presentations.

In this study, we found no statistically significant interaction between diet quality and either PGS to depression or hs-CRP in relation to depressive symptoms. These findings suggest that the association between diet quality and depressive symptoms did not appear to differ materially across levels of genetic risk or low-grade inflammation in this cohort. A prospective analysis from the UK Biobank similarly reported no significant interaction between genetic risk and combined lifestyle factors, including diet, supporting the possibility that diet quality may be relevant to depressive symptoms irrespective of genetic susceptibility [24]. Similarly, the absence of effect modification by hs-CRP suggests that the association between diet quality and depressive symptoms did not vary according to levels of low-grade systemic inflammation. However, these findings should be interpreted cautiously. Although systemic inflammation has been proposed as a potential mediator linking diet quality with depressive symptoms, there remains limited evidence regarding its role as an effect modifier [22].

Several explanations may account for the lack of observed interaction. The pathways linking diet quality with depressive symptoms are likely multifactorial and may not be adequately captured by a single modifier [55]. While inflammation is biologically plausible, hs-CRP represents only one biomarker and may not fully reflect the complexity, chronicity, or tissue-specific nature of inflammatory processes relevant to depression [56]. In addition, participants may have followed specific dietary patterns long before time zero, and this history of dietary exposure could influence their pre-existing inflammatory status (hs-CRP level). Similarly, the depression polygenic score may not capture all relevant genetic or gene-environment pathways underlying susceptibility to diet-related depressive symptoms [57].

In the sex-disaggregated exploratory analysis, the association between diet quality and depressive symptoms remained significant among female participants and no association was observed among males. Females are more likely to develop depression than males [58], and studies have also primarily linked diet to depressive symptoms in females [18, 59]. Several mechanisms have been proposed to account for this disparity, including environmental, behavioural, and biological domains, as well as genetic differences [58, 60]. Biological explanations may include sex differences in hormonal milieu, immune function, stress responsivity, body composition, and genetic architecture relevant to depression [61]. Behavioural and social explanations are also possible, including differences in dietary patterns, food choices, health behaviours, help-seeking, symptom reporting, and exposure to psychosocial stressors [62].

Moreover, evidence shows higher polygenicity for depression in females than in males [60]. However, because the sex interaction was not statistically significant, these sex-specific findings should be interpreted as exploratory.

To our knowledge, this is the first study that examines the role of diet in depression in older people and investigates the effect-modification roles of genetic predisposition and low-grade inflammation. We applied a robust target trial emulation design that accounted for PGS and low-grade inflammation, alongside other known confounders, in a well-characterised cohort of older adults followed over a long period.

However, there are some limitations warranting discussion. First, dietary intake was assessed using a self-reported diet screener, from which we were unable to estimate calorie intake, and which may introduce a recall bias. Participants may have been following a specific dietary pattern for an unknown period before time zero, which could introduce prevalent user bias. Second, although the CES-D 10 is a validated instrument for identifying clinically relevant depressive symptoms, it does not substitute for a clinical diagnosis of depression. Third, despite adjusting for a broad range of potential confounders and examining the robustness of findings to unmeasured confounders by reporting E-values, the role of residual confounding cannot be fully excluded. Fourth, although subgroup and interaction analyses were undertaken, outcome assessment spanned both the ASPREE trial and ASPREE-XT post-trial follow-up; therefore, a residual influence of the original aspirin intervention cannot be fully excluded, despite previous ASPREE-D analyses showing no preventive or long-term benefit of low-dose aspirin for depression. Finally, as our analyses were restricted to participants of European ancestry, the generalisability of our findings to other ancestries is limited.

## Conclusion

High consumption of ultra-processed foods was associated with a higher risk of depressive symptoms, whereas an anti-inflammatory dietary pattern was associated with a lower risk in older adults. We found no clear evidence that these associations differed by genetic susceptibility to depression or low-grade systemic inflammation. These findings suggest that diet quality may be relevant to depressive symptoms across different biological risk profiles. While the observed associations were modest, they may have population-level relevance given the modifiable nature of diet. However, causal interpretation should remain cautious. Overall, these results support the potential importance of diet quality in strategies to promote mental health in later life.

## Supplementary information

Supplementary files

## Data Availability

Researchers interested in accessing the dataset should request to Monash University at https://aspree.org/aus/for-researchers/. The analysis code is available from the corresponding author upon reasonable request.
