# Supplementary material for: Diet quality and depressive symptoms in older adults, assessing the effect modification by genetic predisposition and low-grade inflammation: a target trial emulation
Source: Mol Psychiatry. 2026 Jul 17;31(12):7081–90. doi: 10.1038/s41380-026-03762-6 (PMC13736683; doi:10.1038/s41380-026-03762-6)
Supplement: Supplementary file 1 [file 41380_2026_3762_MOESM1_ESM.docx]

**Supplementary files**

**S. Table S1:** Food frequency questionnaire used in ASPREE longitudinal study of older persons (ALSOP)

| **Food Item** | Never/ rarely | Once or twice a  month | Once or twice a  week | Often or 3-6 times  per week | Every day or several  times a day |
| --- | --- | --- | --- | --- | --- |
| Cheese |  |  |  |  |  |
| Yoghurt |  |  |  |  |  |
| Cream, cream cheese or similar |  |  |  |  |  |
| Ice cream, frozen yoghurt or  other dairy desserts |  |  |  |  |  |
| White bread |  |  |  |  |  |
| Brown/multigrain bread |  |  |  |  |  |
| Breakfast cereal/ oats |  |  |  |  |  |
| Rice |  |  |  |  |  |
| Pasta / noodles |  |  |  |  |  |
| Crackers / savoury biscuits |  |  |  |  |  |
| Fresh fruit |  |  |  |  |  |
| Cooked, tinned or dried fruit |  |  |  |  |  |
| Raw/salad vegetables |  |  |  |  |  |
| Cooked green vegetables |  |  |  |  |  |
| Cooked orange / yellow / red vegetables e.g. carrots, beets |  |  |  |  |  |
| Potatoes |  |  |  |  |  |
| Beans and Legumes  e.g. peas, lentils |  |  |  |  |  |
| Red meat (not corned, pickled or processed) |  |  |  |  |  |
| Chicken, turkey, other poultry |  |  |  |  |  |
| Sausages |  |  |  |  |  |
| Processed meats e.g. bacon,  ham, corned beef or salami |  |  |  |  |  |
| Eggs |  |  |  |  |  |
| Deep fried/battered fish |  |  |  |  |  |
| Salmon, mackerel or other oily fish  not including deep fried fish |  |  |  |  |  |
| White fish,  not including deep fried fish |  |  |  |  |  |
| Tinned fish e.g. canned salmon,  tuna, sardines |  |  |  |  |  |
| Other seafood e.g. prawns or  Shellfish |  |  |  |  |  |
| Nuts |  |  |  |  |  |
| Potato chips or similar |  |  |  |  |  |
| Sweet biscuits/ cakes |  |  |  |  |  |
| Dark Chocolate |  |  |  |  |  |
| Milk Chocolate |  |  |  |  |  |
| Lollies or other sweets |  |  |  |  |  |
| Hamburgers, pizza or 'fast' food |  |  |  |  |  |
| Meat pies, sausage rolls etc |  |  |  |  |  |
| **Drinks** | Never/ rarely | Once per week or  less | Several times a week | 1-2 times a day | 3 or more times/day |
| Full-cream milk |  |  |  |  |  |
| Low-fat or skim milk |  |  |  |  |  |
| Soy or other non-dairy milk |  |  |  |  |  |
| Water |  |  |  |  |  |
| Coffee |  |  |  |  |  |
| Tea |  |  |  |  |  |
| Malt drinks e.g. Milo or Horlicks |  |  |  |  |  |
| Hot chocolate |  |  |  |  |  |
| Fruit juice |  |  |  |  |  |
| Cordial |  |  |  |  |  |
| Soft Drink e.g. reguar Coke |  |  |  |  |  |
| Diet Soft drink e.g. diet Coke |  |  |  |  |  |
| Supplement drink  e.g. Ensure or Sustagen |  |  |  |  |  |
| Herbal tea |  |  |  |  |  |
| What was the most common cooking oil used in your household? | | | |  |  |
| Olive oil | | | | | |
| Butter | | | | | |
| Other vegetable oil | | | | | |
| Don’t know | | | | | |
| Did you regularly add salt to your cooking? | | | | Yes | No |
| Did you regularly add salt to your food at the table? | | | | Yes | No |
| Do you currently take any of the following? | | | No | Yes, occasionally | Yes, every day |
| Fish oil | | |  |  |  |
| Glucosamine | | |  |  |  |
| Coenzyme Q10 | | |  |  |  |
| Zinc supplements | | |  |  |  |
| Calcium supplements | | |  |  |  |
| Multivitamin | | |  |  |  |
| Vitamin B (including Thiamine) | | |  |  |  |
| Vit C | | |  |  |  |
| Vit D | | |  |  |  |
| Vit E | | |  |  |  |
| Gingko Biloba | | |  |  |  |
| Chinese or herbal medicines | | |  |  |  |
| Other supplements, herbal or complementary medicines | | |  |  |  |

**S. Table 2:** The effect modification role of genetic predisposition and hs-CRP in the association between diet quality and depressive symptoms

|  |  | | **Depressive symptoms (CES-D 10≥8)** | |
| --- | --- | --- | --- | --- |
|  |  |  | RR (95%CI) | P-value |
| ***Diet-genetic interaction** | UPF | | | |
|  | UPF consumption | High | 1.13 (1.05-1.23) | 0.002 |
|  |  | Low | 1.00 |  |
|  | PGS | | 1.23 (1.18-1.28) | <0.001 |
|  | UPF x PGS |  | **0.95 (0.87-1.03)** | **0.204** |
|  | DIS | | | |
|  | DIS | Anti-inflammatory | 0.92 (0.85-0.99) | 0.029 |
|  |  | Pro-inflammatory | 1.00 |  |
|  | PGS | | 1.16 (1.10-1.22) | <0.001 |
|  | DIS x PGS |  | **1.07 (1.00-1.16)** | **0.056** |
| ****Interaction between diet and low-grade inflammation** | UPF | | | |
|  | UPF consumption | High | 1.10 (1.00-1.21) | 0.054 |
|  |  | Low | 1.00 |  |
|  | Hs-CRP | | 1.08 (1.04-1.13) | <0.001 |
|  | UPF x hs-CRP |  | **1.02 (0.94-1.10)** | **0.691** |
|  | DIS | | | |
|  | DIS | Anti-inflammatory | 0.97 (0.89-1.06) | 0.470 |
|  |  | Pro-inflammatory | 1.00 |  |
|  | CRP | | 1.12 (1.06-1.18) | <0.001 |
|  | DIS x hs-CRP |  | **0.95 (0.89-1.02)** | **0.171** |

- x: Interaction
- PGS: standardised polygenic score
- *Interaction between diet quality (UPF consumption and dietary inflammation score (DIS) and standardised polygenic score (PGS) for risk of depressive symptoms
- **Interaction between diet quality (UPF consumption and dietary inflammation score (DIS) and log hs-CRP (high sensitivity C-reactive protein) for risk of depressive symptoms
- Covariates in the main analysis were accounted for, except for variables used in the interaction.

**S. Table 3:** Interaction analysis using categorical PGS and hs-CRP as effect modifiers

|  |  | | | **Depressive symptoms (CES-D 10≥8)** | |
| --- | --- | --- | --- | --- | --- |
|  |  |  |  | RR (95%CI) | P-value |
| ***Diet-genetic interaction** | UPF | | | | |
|  | UPF consumption (high vs low) | | | 1.25 (1.03-1.53) | 0.025 |
|  | PGS | | High | 1.74 (1.51-2.00) | <0.001 |
|  |  | | Medium | 1.36(1.20-1.54) | <0.001 |
|  | UPF x PGS | | High x high | **0.91 (0.70-1.17)** | **0.454** |
|  |  |  | High x medium | **0.87 (0.69-1.08)** | **0.212** |
|  | DIS | | | | |
|  | DIS | Anti-inflammatory vs pro-inflammatory | | 0.96 (0.79-1.15) | 0.630 |
|  | PGS | | High | 1.54 (1.29-1.84) | <0.001 |
|  |  |  | Medium | 1.35 (1.16-1.57) | <0.001 |
|  | DIS x PGS | | High x anti-inflammatory | **1.13 (0.89-1.44)** | **0.305** |
|  |  |  | Medium x anti-inflammatory | **0.91(0.74-1.12)** | **0.376** |
| ****Interaction between diet and low-grade inflammation** | UPF | | | | |
|  | UPF consumption (high vs low) | | | 1.07 (0.97-1.18) | 0.168 |
|  | Hs-CRP (high vs low) | | | 1.17 (1.07-1.29) | 0.001 |
|  | UPF x hs-CRP | | High x high | **1.10 (0.92-1.31)** | **0.287** |
|  | DIS | | | | |
|  | DIS | | Anti-inflammatory vs pro-inflammatory | 0.97 (0.89-1.06) | 0.496 |
|  | Hs-CRP (high vs low) | | | 1.11 (1.05-1.17) | <0.001 |
|  | DIS x hs-CRP | | High x anti-inflammatory | **0.96 (0.90-1.03)** | **0.242** |

- x: Interaction
- PGS: high (Q5), medium (Q2-Q4) and low (Q1- reference group)
- Hs-CRP: high(>3mg/L) and low (≤3mg/L- reference group)
- X: interaction
- Covariates in the main analysis were accounted for, except for variables used in the interaction.

**S. Table 4:** Sex-disaggregated analysis showing the association between diet quality and depressive symptoms, adjusting for PGS and hs-CRP

| Male | |  | |
| --- | --- | --- | --- |
| Diet quality | | Depressive symptoms (CES-D 10≥8) | |
|  |  | RR (95%CI) | P-value |
| UPF consumption | High | 1.08 (0.96-1.23) | 0.209 |
|  | Low | 1.00 |  |
| DIS | Anti-inflammatory | 0.98 (0.87-1.10) | 0.713 |
|  | Pro-inflammatory | 1.00 |  |
| Female |  |  |  |
| UPF consumption | High | 1.20 (1.08-1.33) | 0.001 |
|  | Low | 1.00 |  |
| DIS | Anti-inflammatory | 0.90 (0.82-0.99) | 0.030 |
|  | Pro-inflammatory | 1.00 |  |

**S. Table 5:** Sex-disaggregated analyses exploring the effect modification of genetic predisposition and low-grade inflammation in the association between diet quality and depressive symptoms

| Male |  | | | Depressive symptoms (CES-D 10≥8) | |
| --- | --- | --- | --- | --- | --- |
|  |  |  |  | RR (95%CI) | P-value |
|  | Diet-genetic interaction | UPF | High | 1.14 (1.02-1.29) | 0.027 |
|  |  | PGS | | 1.33 (1.24-1.43) | <0.001 |
|  |  | High x PGS | | 0.87 (0.77-0.98) | 0.027 |
|  |  | DIS | Anti-inflammatory | 0.95 (0.84-1.07) | 0.383 |
|  |  | PGS | | 1.18 (1.09-1.28) | <0.001 |
|  |  | Anti-inflammatory x PGS | | 1.17 (1.04-1.31) | 0.009 |
|  | Diet x hs-CRP | UPF | High | 1.09 (0.96-1.25) | 0.184 |
|  |  | Hs-CRP | | 1.07 (1.00-1.14) | 0.049 |
|  |  | High x hs-CRP | | 1.04 (0.93-1.15) | 0.522 |
|  |  | DIS | Anti-inflammatory | 1.00 (0.88-1.15) | 0.948 |
|  |  | Hs-CRP | | 1.10 (1.02-1.19) | 0.015 |
|  |  | Anti-inflammatory x hs-CRP | | 0.95 (0.86-1.06) | 0.398 |
| Female | Diet-genetic interaction | UPF | High | 1.16 (1.05-1.29) | 0.004 |
|  |  | PGS | | 1.17 (1.11-1.23) | <0.001 |
|  |  | High x PGS | | 1.00(0.91-1.10) | 0.984 |
|  |  | DIS | Anti-inflammatory | 0.93 (0.85-1.01) | 0.096 |
|  |  | PGS | | 1.18(1.11-1.26) | <0.001 |
|  |  | DIS x PGS |  | 0.98 (0.90-1.07) | 0.696 |
|  | Diet x hs-CRP | UPF | High | 1.14 (1.00-1.29) | 0.046 |
|  |  | Hs-CRP | | 1.08 (1.03-1.13) | 0.002 |
|  |  | High x CRP | | 1.01 (0.91-1.11) | 0.888 |
|  |  | DIS | Anti-inflammatory | 0.94(0.85-1.05) | 0.297 |
|  |  | Hs-CRP | | 1.08 (1.01-1.15) | 0.016 |
|  |  | Anti-inflammatory x hs-CRP | | 0.99 (0.91-1.08) | 0.814 |

**S. Table 6:** Stratified analysis by aspirin treatment (aspirin vs placebo)

| **Exposure categories** | **Depressive symptoms (CES-D 10 ≥8)** | | | |
| --- | --- | --- | --- | --- |
|  | **Aspirin** | | **Placebo** | |
|  | RR (95% CI) | p-value | RR (95% CI) | p-value |
| **UPF consumption** | | | | |
| High | 1.17 (1.03-1.32) | 0.016 | 1.10 (0.97-1.25) | 0.145 |
| Low | 1.00 |  | 1.00 |  |
| **DIS** | | | | |
| Anti-inflammatory | 0.89 (0.80-1.00) | 0.052 | 0.98 (0.88-1.10) | 0.786 |
| Pro-inflammatory | 1.00 |  | 1.00 |  |
| **Interaction** |  | | |  |
| UPF x aspirin | 0.94 (0.79-1.12) | | | 0.510 |
| DIS x aspirin | 1.10 (0.94-1.29) | | | 0.234 |

**S. Table 7:** Association between diet quality and depressive symptoms, adjusting for PGS and hs-CRP after excluding participants with depressive symptoms (CES-D 10 cut-off ≥8) and taking antidepressants/antipsychotics at time zero.

| Diet quality | | Depressive symptoms (CES-D 10≥8) | |
| --- | --- | --- | --- |
|  |  | RR (95%CI) | P-value |
| **Excluding participants with depressive symptoms at time 0** | | | |
| UPF consumption | High | 1.10 (1.00-1.23) | 0.054 |
|  | Low | 1.00 |  |
| DIS | Anti-inflammatory | 0.93 (0.85-1.01) | 0.099 |
|  | Pro-inflammatory | 1.00 |  |
| **Excluding participants taking antidepressants/antipsychotics at time 0** | | | |
| UPF consumption | High | 1.12 (1.02-1.24) | 0.014 |
|  | Low | 1.00 |  |
| DIS | Anti-inflammatory | 0.91 (0.84-0.99) | 0.029 |
|  | Pro-inflammatory | 1.00 |  |

**S. Table 8:** Association between diet quality and depressive symptoms, adjusting for PGS and hs-CRP and effect-modification analysis using CES-D 10 cut-off ≥10

| Diet quality | | Depressive symptoms (CES-D 10≥10) | |
| --- | --- | --- | --- |
|  |  | RR (95%CI) | P-value |
| **Main effect** | | | |
| UPF consumption | High | 1.11 (1.00-1.24) | 0.046 |
|  | Low | 1.00 |  |
| DIS | Anti-inflammatory | 0.93 (0.84-1.02) | 0.143 |
|  | Pro-inflammatory | 1.00 |  |
| **Interaction effect** | | | |
| UPF x PGS | | 0.95 (0.85-1.06) | 0.343 |
| UPF x hs-CRP | | 0.99 (0.90-1.09) | 0.864 |
| DIS x PGS | | 1.08(0.98-1.19) | 0.117 |
| DIS x hs-CRP | | 0.94 (0.86-1.03) | 0.175 |

PGS: standardised polygenic score, hs-CRP: log high-sensitivity C-reactive protein

**S. Figure 1:** ASPREE and ASPREE-XT study timeline showing CES-D follow-up data collection periods included in the current analysis

**S. Figure 2:** Directed acyclic graphs (DAGs) showing potential confounders.

**S. Figure 3:** Balance plots of diet quality indicators (UPF and DIS) showing before and after IPTW.

**S. Figure 4**: Plot showing standardised mean difference (SMD) for covariates at time zero

**a)**

**b)**

**c)**

**d)**

**S. Figure 5:** Effect modification of polygenic score to depression (a and b) and low-grade inflammation (c and d) in the association between diet quality and depressive symptoms.

**S. Figure 6:** Stratified analysis showing the association between diet quality and depressive symptoms by PGS category

DIS: Dietary inflammation score, anti-inf: anti-inflammatory, pro-inf: pro-inflammatory, UPF: ultra-processed food. All covariates in the main analysis were included in the IPTW, except for the stratification variable (PGS).

**S. Figure 7:** Association between the combination of genetic risk and diet quality and risk of depressive symptoms

UPF: Ultra-processed food, DIS: Dietary inflammation score, anti-inf: anti-inflammatory diet, pro-inf: pro-inflammatory diet. All covariates accounted for in the main model, except PGS, were included in IPTW.

As shown in **S. Figure 7**, using participants with low genetic risk and low UPF consumption as the reference group, those with high genetic risk and high UPF consumption had the highest risk of depressive symptoms (RR: 1.72, 95% CI: 1.43-2.07). Conversely, participants with low genetic risk and an anti-inflammatory diet showed the greatest reduction in depressive symptoms compared to those with high genetic risk and a pro-inflammatory diet (RR: 0.65, 95% CI: 0.54-0.78).

**a)**

**b)**

**c)**

**d)**

**S. Figure 8:** Effect modification of polygenic score to depression in the association between diet quality and depressive symptoms by sex: male (**a** and **b**) and female (**c** and **d**).
